# Emotional tone in clinical high risk for psychosis: novel insights from a natural language analysis approach

**DOI:** 10.3389/fpsyt.2024.1389597

**Published:** 2024-05-13

**Authors:** Gabrielle M. Olson, Katherine S. F. Damme, Henry R. Cowan, Luz Maria Alliende, Vijay A. Mittal

**Affiliations:** ^1^ Department of Psychology, Northwestern University, Evanston, IL, United States; ^2^ Institute for Innovations in Developmental Sciences (DevSci), Northwestern University, Evanston and Chicago, IL, United States; ^3^ Department of Psychiatry, Northwestern University, Chicago, IL, United States; ^4^ Department of Psychiatry and Behavioral Health, The Ohio State University, Columbus, OH, United States; ^5^ Department of Psychology, Michigan State University, East Lansing, MI, United States; ^6^ Medical Social Sciences, Northwestern University, Chicago, IL, United States; ^7^ Institute for Policy Research (IPR), Northwestern University, Chicago, IL, United States

**Keywords:** emotion, language, natural language processing (NLP), clinical high risk (CHR) for psychosis, prodrome

## Abstract

**Background:**

Individuals at clinical high risk (CHR) for psychosis experience subtle emotional disturbances that are traditionally difficult to assess, but natural language processing (NLP) methods may provide novel insight into these symptoms. We predicted that CHR individuals would express more negative emotionality and less emotional language when compared to controls. We also examined associations with symptomatology.

**Methods:**

Participants included 49 CHR individuals and 42 healthy controls who completed a semi-structured narrative interview. Interview transcripts were analyzed using Linguistic Inquiry and Word Count (LIWC) to assess the emotional tone of the language (tone -the ratio of negative to positive language) and count positive/negative words used. Participants also completed clinical symptom assessments to determine CHR status and characterize symptoms (i.e., positive and negative symptom domains).

**Results:**

The CHR group had more negative emotional tone compared to healthy controls (*t*=2.676, *p*=.009), which related to more severe positive symptoms (*r^2^* =.323, *p*=.013). The percentages of positive and negative words did not differ between groups (*p*’s>.05).

**Conclusions:**

Language analyses provided accessible, ecologically valid insight into affective dysfunction and psychosis risk symptoms. Natural language processing analyses unmasked differences in language for CHR that captured language tendencies that were more nuanced than the words that are chosen.

## Introduction

During the clinical high-risk (CHR) period, individuals face a heightened risk of fully converting to a psychotic disorder (1). The period is characterized by attenuated symptoms that precede the onset of psychosis and may emerge with impairment, distress, and functional decline that increase as illness progression continues (2). Studying subthreshold symptoms of psychosis in the CHR period may predict psychosis onset and support a more comprehensive clinical perspective of a patient alongside other risk factors, such as family history of a psychotic disorder (3).

Emotional expression abnormalities in individuals at clinical high risk for psychosis are often subtle but may have substantial impact on social functioning and provide insight into emerging symptoms. However, assessing these deficits is challenging and costly, requiring the resources and time to produce reliable, trained clinical raters. Natural language processing (NLP) methods provide a promising new direction for assessment and more sensitive classification of emotional expression deficits in CHR individuals. Critically, these NLP methods are automated, requiring little training and low costs to implement. NLP analysis of speech has grown in popularity in schizophrenia research and has entered CHR research as well, tracking language differences in CHR individuals that otherwise may have gone unnoticed (4). Although this automated approach has not been implemented in emotional expression through language, similar work has automated assessment of facial expression evaluation and has demonstrated similar potential to streamline laborious approaches (5). Automated approaches can also capture effects than manual coders miss. For example, in a study of facial expression automated approaches highlighted how rather than CHR youth showing decreased expression overall, as suggested by clinical raters, there could be a pattern of decreased positive and increased negative facial expressions in individuals at CHR (5) and those with a schizophrenia diagnosis (6). Similarly, NLP methods may increase the sensitivity to language emotional expression deficits in CHR individuals and provide insight into the nature of these deficits for early detection and intervention.

Blunted affect and emotional expression abnormalities are a prominent negative symptom of schizophrenia (6, 7). However, the intensity of these differences may be decreased along the psychosis risk spectrum, making the absence of emotional expression difficult to detect. Although previous research has not used emotional language to examine emotional expression abnormalities, we can draw on a substantial literature regarding emotional expression deficits using facial expressions to form hypotheses about the types of deficits that we expect to see in the prodromal period. In clinical assessment, individuals with psychosis show less emotion through vocal and facial expressions (6). Despite this decreased overall clinical rating of facial expression, studies of muscle underlying facial expression demonstrate that people with psychosis have imperceptible, coherent facial expressions (6) but express less positive emotion when viewing positive images (8). Individuals at CHR exhibited a similar pattern of facial expression to individuals with a schizophrenia diagnosis, showing blunted positive emotion and increased negative emotion (5) and blunted positive expression when viewing positive stimuli (9). What to expect from emotional expression through language is unclear, but from facial expression literature, it is possible that emotional expression will parallel facial expression with a pattern of less positive and more negative emotionality. However, emotional expression through language might provide unique information beyond looking at facial expression alone. As a result, novel automated approaches may increase our sensitivity to affective differences in emotional expression.

The words that patients use may be a powerful tool to gain insight into subtle emerging deficits. In fact, language has offered new insights into other symptom dimensions in studies of patients with schizophrenia. In a study of schizophrenia patients, lexical analysis showed that regardless of clinical anhedonia, all patients with schizophrenia exhibited more negative emotion and normative positive emotion than controls in speech samples elicited from an interview (10). Minor et al. (11) expanded upon this work to include all symptoms of schizophrenia and discovered that use of negative emotion words, specifically related to anger, predicted higher symptom severity. This work has not been expanded to examine the insight that language use might provide into emotional expression differences in the CHR population prior to psychosis onset, so it is unclear if these changes in language may be a sensitive corollary to detect emerging affective difficulties. In CHR populations, for whom attenuated emotional expression deficits may be more difficult to quantify, individuals may show comparable language patterns through greater levels of negative emotion in speech.

Other measures of emotional expression deficits are impacted in CHR individuals and predict worsening clinical course or conversion to a formal psychosis diagnosis. Blunted expression of emotion in the CHR period has been found to strongly predict psychosis conversion and more severe negative symptoms in schizophrenia (12). Taken together this work highlights the potential for early emotional expression deficits to predict worsening clinical course and conversion. However, this has not been examined in the emotional expression of language. Indeed, there is great potential clinical utility of NLP methods that may aid in the detection of subtle emotional expression differences associated in CHR individuals that may have practical and predictive clinical utility in the future. While quantifying emotional expression can be difficult, NLP methods may be useful tools with other clinical measures to capture early variability in emotionality.

Novel, automated approaches towards language analysis aim to simplify and optimize psychosis prediction methods to the level of clinical ratings and beyond (4). With multiple techniques already utilized in psychosis research to investigate linguistic features, such as thought disorder and prediction of clinical outcomes, like suicidal ideation, this work shows potential for the future (4, 13–15). Automated methods can improve language analysis by quickly measuring linguistic features that differ between clinical groups, unlike manual coding that may take months to achieve the same aim. It is important to consider the type of NLP model used for analysis, as they may be able to predict clinical group to varying degrees of success (16). Language usage, including the use of “I” statements and sematic similarity scores of negative words (e.g., anger) and evaluated using NLP tools, predicted suicidality in individuals at CHR (14). In one study, NLP methods accurately predicted who would later convert to psychosis, performing better than trained clinical raters (17). An application of NLP to spoken narrative interviews by individuals with schizophrenia found that their speech differed from those without schizophrenia in being less goal-driven and causal (15). Both Cohen et al. (10) and Minor et al. (11) used lexical analysis to study the nuances of positive and negative emotion in the speech of individuals with schizophrenia and how they were linked to psychosis symptoms. Based on these findings, NLP approaches may improve the sensitivity of early, subtle clinical symptoms. Indeed, word use uncovers information about a variety of psychological behavior (11). Accessing such information in early stages of psychosis can be made possible by incorporating NLP tools, such as lexical analysis or latent semantic analysis.

The current study aims to identify emotional language differences between the CHR population and healthy controls by applying lexical analysis, a method of NLP, to speech obtained from a narrative interview. We also aim to understand the relationship of emotional expression to symptom severity in the CHR population to determine how these language features may provide greater insight into symptom presentation. If the results yield language differences between clinical groups, it is possible that they may be driven by positive or negative symptoms. Furthermore, this would indicate the efficacy of an NLP method applied to emotional word use in this sample, proposing the question of whether it is more effective and accurate than manual coding.

## Materials and methods

### Participants

A total of 91 participants (49 CHR; 42 control participants) were recruited through the Adolescent Development and Preventative Treatment Program (ADAPT). Participants were classified as having a CHR syndrome if they met criteria for an attenuated psychosis syndrome by the Structured Interview for Psychosis-Risk Syndromes (SIPS). Additional control participants were screened for the presence of a psychosis syndrome and family history of psychosis. Control participants were excluded if they had a first-degree relative with a psychotic disorder or a psychosis-risk syndrome (based on the SIPS). All participants were excluded if they had a current psychotic disorder, IQ < 70, and history of neurological disorder, tic disorder, or head injury. A subset of participants also had a comorbid depression diagnosis, 24 in the CHR group and 6 controls. Recruitment methods included train ads, Craigslist, flyers, and social media. This study was approved by the Institutional Review Board at Northwestern University, and all participants provided informed consent. The current study includes new analyses that overlap with the data collected in Cowan et al. (18). For more information on the overlap in the sample and reasons for inclusion and exclusion, see the **Supplementary Material**. In prior analyses Cowan et al. manually coded the narratives for identity themes related to concepts of self, but here we examine emotional expression through language use and submitted these narratives to an automated system.

### Clinical assessments

#### Structured Interview for Psychosis-Risk Syndromes (SIPS)

Participants completed the SIPS, a semi-structured interview that assesses attenuated psychotic symptoms (19). All interviews were conducted by clinical graduate students who made diagnostic decisions under the direct supervision of Vijay A. Mittal. The interrater reliability criterion was Kappa 0.80. Positive symptom severity was used to identify participants in the CHR group, consisting of five categories: unusual thought content/delusional ideas, suspiciousness/persecutory ideas, grandiose ideas, perceptual abnormalities/hallucinations, and disorganized communication. Negative symptoms were also assessed and included six symptom categories: social anhedonia, avolition, expression of emotion, experience of emotions and self, ideational richness, and occupational functioning. Symptom severity is rated from 0-6, with a 6 indicating that a symptom has reached threshold for psychosis. Symptoms rated at a 3-5 are considered subthreshold and classified as a CHR syndrome.

#### Structured Clinical Interview for DSM-IV Axis I Disorders (SCID)

The SCID, a semi-structured interview, was administered to assess for other psychiatric disorders, such as depression and psychosis. SCID diagnoses were used to record the presence of co-morbid mood diagnoses in both the CHR and control peer group and exclude individuals with a formal psychosis spectrum diagnosis (20).

### Task assessments

#### Speech prompt

Participants completed an abbreviated Life Story Interview (21) in which they narrated four personally significant experiences including a self-defining memory, turning point memory, life challenge, and psychosis-spectrum experience (18). The interviews were audio recorded and transcribed prior to analysis. Further details of the interview can be found in Cowan et al. (18). All text across the four interview questions was included. Interviewer and participant transcribed dialogue were separated, and only participant dialogue was analyzed.

#### Language analysis

Interview transcripts were analyzed using the Linguistic Inquiry and Word Count (LIWC), a text analysis program that uses dictionaries to categorize the psychological meanings of words (22). LIWC has been found to be effective in extracting positive and negative emotion words that relate to emotional expressions (23). This analysis utilized the *Affect* basic dictionary and the *Emotional Tone* summary measure. The *tone_pos* and *tone_neg* dictionaries within the *Affect* dictionary are composed of emotion and emotion-related words, like happy, sad, birthday, or funeral, measured as a percentage. The *Emotional Tone* dimension is an algorithm based on the difference between positive and negative emotion words such that a higher score reflects a more positive *Emotional Tone*, and a lower score reflects a more negative *Emotional Tone* (22, 24).

### Analytical strategy

We considered differences between clinical groups for demographics and checked to see if they would impact emotional tone differences. There were no significant differences in the demographic variables of sex, age, race, income, and student status. We examined sex as a potential mediating factor for known sex differences in expression (25, 26) and included it in the final model because it impacted emotional tone differences. A general linear model was used to analyze the differences in emotional language between groups, with clinical status and sex as independent variables and *Emotional Tone* score as the dependent variable. In additional linear models, the same independent variables were analyzed with the percentage of positive and negative emotion words as dependent variables. Linear models were also run to study relationships between tone and symptom severity with SIPS positive and negative symptom scores as the independent variables and *Emotional Tone* score as the dependent variable while controlling for sex.

All analyses were planned *a priori* including prespecified Bonferroni significance thresholds. The total number of comparisons was one planned model for group comparisons (significant if passed Bonferroni correction p<.05), two follow up models parsing *Emotional Tone* into positive and negative (significant if passed Bonferroni correction p<.025) and two attenuated symptom models for within CHR (significant if passed Bonferroni correction p<.025). Follow-up analyses examined the potential role of a depression diagnosis on *Emotional Tone* of language, which did not influence the direction or magnitude of interpreted findings (**Supplementary Material**). Follow up analyses also corrected for the total amount of words used in responses, which did not change the magnitude or direction of current findings (**Supplementary Material**). Differences of group averages (sex or clinical risk group) will be described using a Cohen’s d (*d*) calculation. Relationships defined by two continuous variables will be described by *r^2^*.

## Results

### Participants

Our sample included 91 participants (53.85% female). There were no significant differences in sex between groups (*X^2^* (90)=1.481, *p*=.224). There was also not a significant difference in age between groups (*t*(90)=-1.645, *p*=.104). Clinical risk groups did not significantly differ in the correcting for word count- the total amount of words used in responses (*p*=.399). See **Tables 1**–**3**.

**Table 1:** Demographic Metrics by Group.

		CHR (*n*=49)	CON (*n*=42)	Whole Sample (*n*=91)	Statistic	*p*-value
	Group	53.85%	46.15%	100%	–	–
	SIPS P total- Mean (SD)	9.98 (4.08)	–	–	–	–
	SIPS N total- Mean (SD)	7.04 (5.50)	–	–	–	–
	Word count- Mean (SD)	1864.76 (884.02)	1703.40 (922.68)	1790.29 (900.65)	*t*(90)=0.847	.399
Sex at Birth					*X^2^* (90)=1.481	.224
	Female	46.94%	61.90%	53.85%		
	Male	53.06%	38.10%	46.15%		
Race					*X^2^* (90)=9.076	.106
	First Nations	0%	2.38%	1.10%		
	Asian	14.29%	23.81%	18.68%		
	Black	28.57%	16.67%	23.08%		
	Central/South American	16.33%	2.38%	9.89%		
	White	34.69%	45.24%	39.56%		
	Interracial	6.12%	9.52%	7.69%		
Student					*X^2^* (90)=0.054	.816
	No	30.61%	26.19%	28.57%		
	Yes	69.39%	73.81%	71.43%		
Income					*X^2^* (90)=7.597	.269
	Less than 10,000	10.20%	7.14%	8.79%		
	10,000-19,999	12.24%	2.38%	7.69%		
	20,000-39,999	12.24%	14.29%	13.19%		
	40,000-59,999	14.29%	4.76%	9.89%		
	60,000-99,999	22.45%	28.57%	25.27%		
	100,000 and above	18.37%	33.33%	25.27%		
	Don’t know or refused	10.20%	9.52%	9.89%		

–, not applicable.

**Table 2:** Age and Language Metrics (CHR, *n*=49).

	Mean	StD	Range
Age	20.98	2.74	15-28
Tone	33.07	14.08	10.31-83.20
Tone Positive	2.13	0.69	1.11-4.75
Tone Negative	1.27	0.52	.38-2.70

**Table 3:** Age and Language Metrics (CON, *n*=42).

	Mean	StD	Range
Age	22.10	3.59	16-30
Tone	40.44	16.29	3.60-78.68
Tone Positive	2.45	0.84	.85-4.68
Tone Negative	1.17	0.67	.28-3.11

### Aim 1a: group differences in overall emotional tone

#### Emotional tone

**Figure 1:**
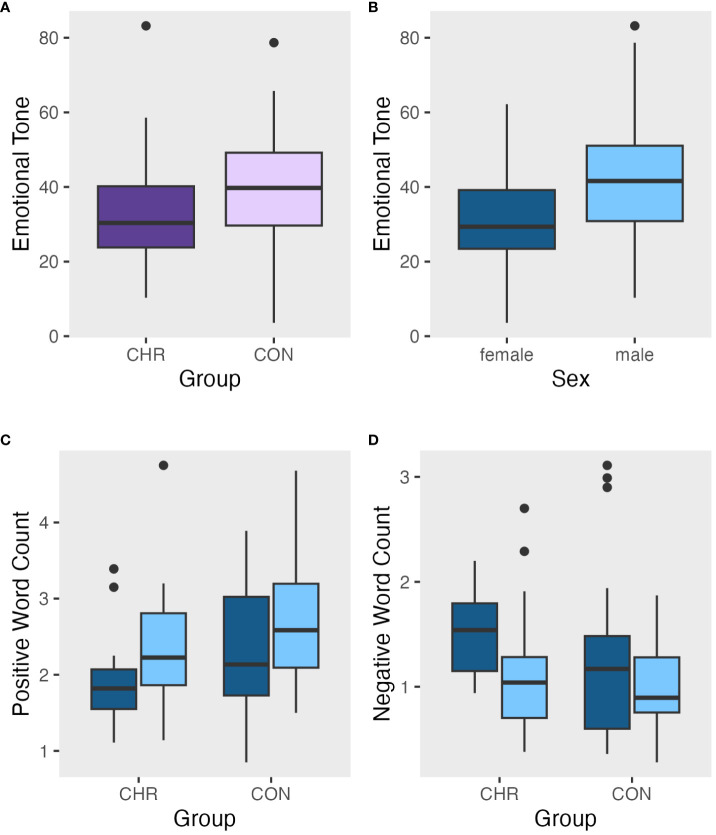
Metrics of emotional tone and word usage: **(A)** Emotional tone by clinical risk group, **(B)** Emotional tone by sex, **(C)** Positive word count by group and sex, **(D)** Negative word count by group and sex. For A and B, a higher emotional tone score means a more positive tone.

Group differences in emotional tone were compared using a general linear model of the average *Emotional Tone* score across clinical groups and sex. There was a significant main effect of clinical group (*t*(90)=2.676, *p*=.009, *d*=0.479) such that the transcripts of individuals at CHR had a lower *Emotional Tone* score (*M*=33.1, *StD*=14.1), meaning a greater proportion of negative emotionally-valenced words, compared to peers (*M*=40.4, *StD*=16.3), **Figure 1A**. There was a significant main effect of sex (*t*(90)=3.277, *p*=.002, *d=*0.683) such that female participants’ transcripts had a lower (*M*=31.8, *StD*=13.8) *Emotional Tone* score compared to their male counterparts (*M*=41.9, *StD*=15.7), **Figure 1B**. There was no significant clinical group by sex interaction (*p*=.523). See **Table 4**.

**Table 4:** Aim 1a Results: Emotional Tone (Whole Sample).

Model parameter	Statistic	*d* (effect size)	*p*-value
Group	*t*(90) = 2.676	0.479	.009
Sex	*t*(90) = 3.277	0.683	.002
Group x Sex	*t*(90) = -.641	–	.523

–, not applicable.

### Aim 1b: group differences in positive and negative emotional tone

#### Positive word count

Group differences in positive tone were compared using a general linear model of the average number of positive emotion words across clinical groups and sex. There was no significant clinical group difference (*p*=.057). There was a non-significant main effect of sex (*t*=2.076, *p*=.041, *d*=0.473) that did not survive correction for multiple comparison, **Figure 1C**. There was no significant clinical group by sex interaction (*p*=.882). See **Supplementary Table 1** for all model details.

#### Negative word count

Group differences in negative tone were compared using a general linear model of the average number of negative emotion words across clinical groups and sex. There was no significant clinical group difference (*p*=.206). There was a significant main effect of sex (*t*(90)=-2.496*, p=*.014, *d=*.602) such that female participants’ transcripts had a higher negative tone score (*M*=1.38, *StD*=.63), meaning more negative emotion words, than male participants’ transcripts (*M*=1.04, *StD=*.49), **Figure 1D**. There was no significant clinical group by sex interaction (*p*=.660). See **Supplementary Table 2** for all model details.

### Aim 2: emotional tone related to symptoms

#### Attenuated positive symptoms

**Figure 2:**
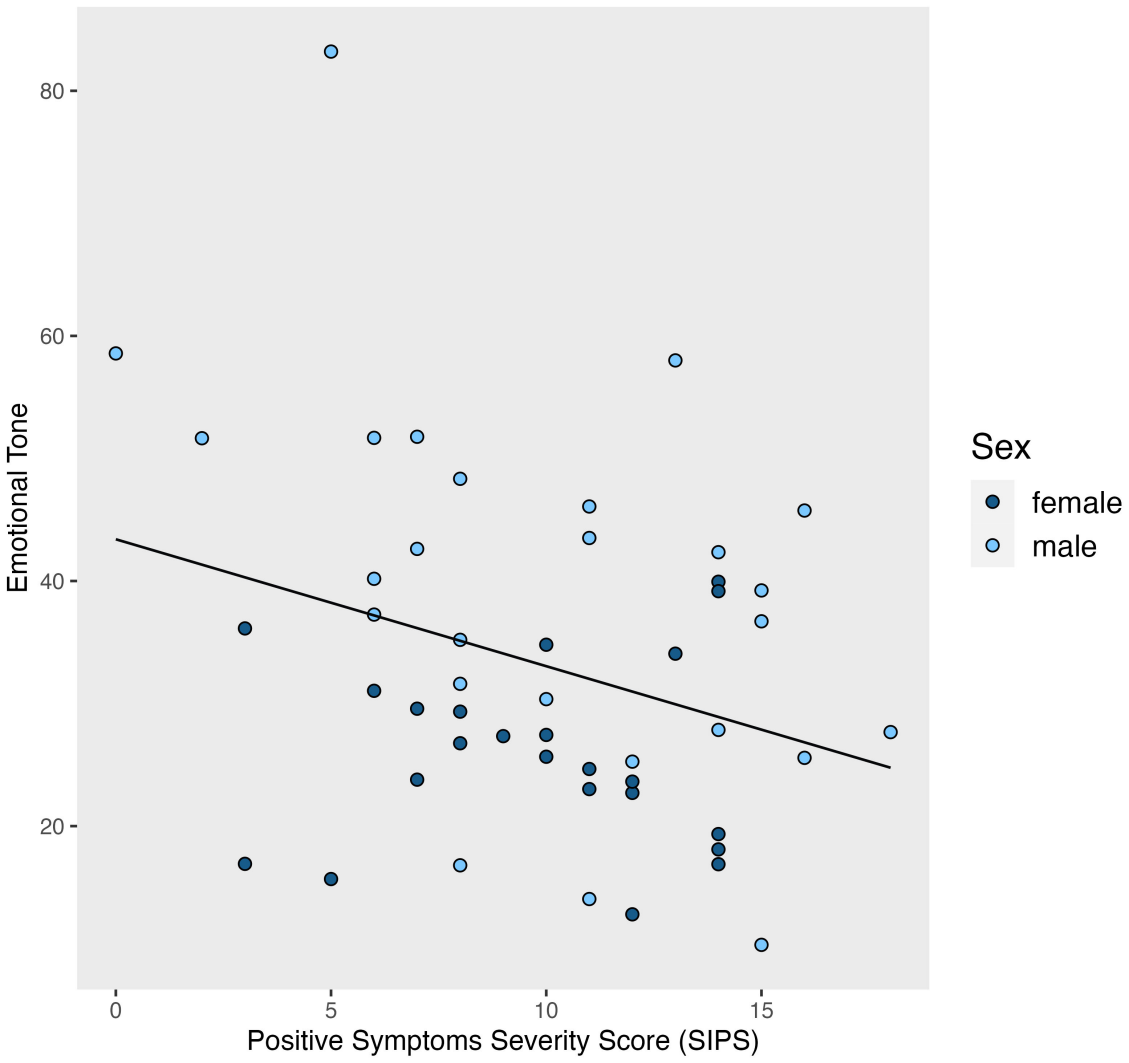
Emotional tone by positive symptom severity score. A higher emotional tone score means a more positive tone.

A general linear model estimated the linear relationship of *Emotional Tone* composite score across SIPS positive attenuated symptom scores while accounting for differences in sex within the CHR group. Lower *Emotional Tone* scores were related to attenuated positive symptoms on the SIPS (*t*(90)=-2.575, *p*=.013, *r^2^=*.323). See **Figure 2**.

#### Attenuated negative symptoms

A general linear model estimated the relationship of *Emotional Tone* composite score across SIPS negative attenuated symptom scores while accounting for the differences in sex within the CHR group. There was no significant relationship (*t*(90)=-1.137, *p*=.262, *r* ^2^=.247).

## Discussion

Individuals at CHR used language with a more negative emotional tone, operationalized as a higher ratio of negative to positive words used. A simple count (measured as a percentage) of negative and positive words did not produce a main effect between clinical groups, signaling the utility of the more dimensional *Emotional Tone* summary variable in LIWC. It also seems that these results are not driven by blunted emotional expression in the CHR group. Individuals showing more negative emotion bias in their emotional tone had greater positive symptom severity, despite expectations that this would reflect negative symptom severity. Taken together, these findings emphasize the benefit of using NLP to unmask nuanced disturbances in emotional expression through language. It is the quality not the quantity of emotional language that distinguishes CHR individuals from peers and along a dimension of positive symptom severity.

### Group differences in *Emotional Tone*

Despite significant group differences in overall emotional tone as measured by the summary score, specific effects were not observed for positive or negative words alone. This finding, which was contrary to our hypotheses, highlights the importance of using more complex metrics to capture emotional word choice. To fully evaluate emotional language in the CHR population, it is not enough to only consider one type of emotion in analyses or overall emotional expression but rather the relation between different forms of affective tone. While LIWC emotional tone metrics are based on predefined dictionaries and not as complex as other NLP techniques, the *Emotional Tone* summary variable accounts for the differences between the word counts in individual narratives and bases the scores on the standardized scores of comparison samples (22). Our results show that this variable was able to interpret differences between CHR and HC groups in specific word usage and structure, obtaining a more comprehensive perspective of their emotional language.

Higher positive symptom severity was related to a more negative emotional tone in individuals at CHR. This feature of linguistic expression may not be adequately captured by any classic measure of symptom assessment but still present when people at CHR are producing language in a clinical assessment setting. This finding is similar to a previous finding using trained raters to code emotional tone (18), yet the present NLP analyses quantified differences in emotional expression without added work, training, or equipment. As such, this method has potential as a highly accessible tool to assess subtle emotional expression. Psychosis research has found that higher negative affect predicts higher positive symptoms (27, 28). Additionally, positive symptoms may be triggered by a stressful life event, the reaction to which is exacerbated by a more negative emotional bias of the situation (29). Developing NLP markers for clinical use requires careful integration of existing knowledge, empirical research, and the replication of predictive models as well as concern for ethical issues (30, 31). Adding NLP methods to clinical assessments in CHR may offer further insight into positive symptom presentation when emotional expression is more challenging to assess.

### Emotional expression and blunting

When emotional tone was measured as a summary score between positive and negative words, CHR individuals showed consistent results with past literature on emotional expressivity in facial features. Greater bias toward negative emotional tone in language is consistent with facial expressions literature in CHR (5, 9) and schizophrenia populations (6, 8) that finds less positive emotional and greater negative emotional expression. There may be a general pattern of a shift towards less positive and more negative emotional expression in multiple modalities in the CHR period before the onset of emotional blunting (6), which may point to an increased risk of psychosis conversion (12). While we did not find high levels of emotional blunting comparable to the severity in schizophrenia, there is still evidence of a spectrum of emotional deficits starting in the CHR period. Also of note, although there was a significant sex difference (as expected; 25, 26), this difference did not diminish the impact of clinical group on emotional tone.

### Further NLP methods

To expand upon this work, more advanced NLP methods that have already been utilized in schizophrenia research may capture additional features of emotional language in the CHR period. For example, latent semantic analysis has detected the decline of semantic coherence in schizophrenia (32) and accurately predicted conversion to psychosis through free speech (17). In studying syntactic disturbances, psychosis onset prediction was improved by using part-of-speech tagging to show lower possessive pronoun usage (33). Our results obtained through lexical analysis support the application of NLP methods to the domain of emotional disturbances in individuals at CHR. Clinical assessment of individuals at CHR traditionally focus on the prevalence of positive or negative emotions, but rarely is the ratio of positive to negative emotions considered.

### Limitations and future directions

Certain limitations exist in this study that provide several future directions for research. The sample sizes were relatively small, and the present results would benefit from replication in larger sample sizes. There are also methodological limitations associated with LIWC, as its analyses originate from the frequencies of words categorized by dictionaries (22). This presents the challenge of labeling words with varied contexts and ambiguous meanings that may inaccurately add or subtract from word counts and summary scores. For example, the phrases “I love you” and “I don’t love you” would have equal positive word counts due to the inclusion of “love,” which would be represented as similar overall *Emotional Tone* scores. A word like “suck” would count as a negative emotion even in the context of sucking on a lollipop. This is a known issue in the field, and contextual embedding can improve the understanding of social discourse in language (34). Moreover, it is important to note there is language variation rooted in individual identities (e.g., sex, socioeconomic status, culture) (31, 35–37). There is still a benefit to including human ratings of language along with automated methods of analysis, as both provide unique strengths and potential usage for clinical work (38). Future research should expand upon this to finetune facets of identity that could impact how people are speaking about emotion. For example, training models using language production from youth with similar cultural backgrounds could account for some of these nuances.

CHR status is defined by the presence of attenuated positive symptoms, but this recruitment strategy may not be ideal for understanding the influence of negative symptoms, including potential relationships between attenuated negative symptoms and emotional language. Future research should also recruit participants who are experiencing more negative symptoms to examine the relation of emotional tone to symptom severity. A greater focus on negative symptoms to determine clinical group status could yield significant correlations with emotional tone. Finally, exploratory analyses were conducted in this study on whether emotional language could be affected by depression, which did not affect the magnitude or direction of current findings (**Supplementary Material**). However, emotional expression abnormalities may yet be linked to affective symptom dimensions in individuals at CHR. Future research should examine emotional symptom dimensions (e.g., depression, anxiety) as a potential mediator of language use in emotional expression.

## Conclusion

In this investigation of emotional disturbances in individuals at CHR, distinct emotional language differences were unmasked by NLP analyses. The summary dimension of *Emotional Tone* in LIWC identified nuances in word usage that differentiated clinical groups, building upon emotion word counts to detect additional variation in participant language. NLP methods beyond simple lexical analysis may allow us to capture small differences in emotional expressivity but more importantly to hone into the relation between different forms of affect. NLP is a promising avenue for psychosis risk research and one that continues to benefit from technological advancements in psychology.

## Data availability statement

The raw data supporting the conclusions of this article will be made available by the authors, without undue reservation.

## Ethics statement

The studies involving humans were approved by Northwestern University Institutional Review Board. The studies were conducted in accordance with the local legislation and institutional requirements. Written informed consent for participation in this study was provided by the participants or participants' legal guardian/next of kin.

## Author contributions

GO: Conceptualization, Formal analysis, Visualization, Writing – original draft, Writing – review & editing, Methodology. KD: Conceptualization, Formal analysis, Supervision, Visualization, Writing – original draft, Writing – review & editing, Methodology. HC: Data curation, Investigation, Resources, Writing – review & editing. LA: Methodology, Writing – review & editing. VM: Conceptualization, Funding acquisition, Investigation, Methodology, Project administration, Writing – review & editing, Supervision, Resources.
