# Supplementary material for: Emotional tone in clinical high risk for psychosis: novel insights from a natural language analysis approach
Source: Front Psychiatry. 2024 May 13;15:1389597. doi: 10.3389/fpsyt.2024.1389597 (PMC11128650; doi:10.3389/fpsyt.2024.1389597)
Supplement: Supplementary file 1 [file DataSheet_1.docx]

Supplementary Material

**I. Sample Overlap**

The current study utilized an overlapping sample of participants to Cowan et al. (2023). The sample differences are due to the latter paper including different versions of the Life Story Interview, some with 3 questions and some with 4 questions. The current study only used versions with 4 questions for consistency between participants. Additionally, not all data had been entered at the time of publication for Cowan et al. (2023). As a result, the current study includes some individuals not previously available for publication. For CHR participants, there was 97.96% overlap between the current study (*n*=49) and Cowan et al. (2023) (*n*=49). One participant was removed due to having the 3-question interview, and one was added due to new data. For control participants, there was 57.14% overlap between the current study (*n*=42) and Cowan et al. (2023) (*n*=52). 14 participants were removed due to having the 3-question interview, and four were added due to new data.

**II. Supplementary Analyses**

In a follow-up, exploratory analysis, we examined the potential influence of depression diagnosis on the results, as initial analyses did not account for diagnosis differences in the CHR group. In additional linear models, depression was included as a mediating factor using current or past diagnoses of major depressive disorder (MDD) as assessed by the SCID. Group differences in emotional tone were compared using a general linear model of the average emotional tone score across clinical group, sex, and MDD diagnosis. There continued to be a significant main effect of clinical group (*X^2^*(90)*=*10.798,  *p*=.008), but there were no other main or interaction effects.

Clinical groups did not differ in total word count of the transcripts (*t*(90)=.848, *p*=.399), and we corrected for word count in additional linear models. For group differences in emotional tone, there continued to be a significant main effect of clinical group (*t*(90)=2.516, *p*=.014) and sex (*t*(90)=3.154, *p*=.002). For negative and positive word count, there continued to be no significant main effect of clinical group. There was still a significant main effect of sex for negative word count (*t*(90)=-2.508, *p*=.014) but not for positive word count (*t*(90)=1.936, *p*=.056). Additionally, lower emotional tone scores continued to be significantly related to higher positive symptom scores (*t*(90)=-2.438, *p*=.019, *r^2^*=.352).

**III. Supplementary Tables**

Table 1: Aim 1b Results: Positive Word Count (Whole Sample)

| Model parameter | Statistic | Effect size | *p*-value |
| --- | --- | --- | --- |
| Group | *t*(90) = 1.926 | 0.416 | 0.057 |
| Sex | *t*(90) = 2.076 | 0.473 | 0.041 |
| Group x Sex | *t*(90) = -0.149 | - | 0.882 |

Table 2: Aim 1b Results: Negative Word Count (Whole Sample)

| Model parameter | Statistic | Effect size | *p*-value |
| --- | --- | --- | --- |
| Group | *t*(90) = -1.276 | 0.167 | 0.206 |
| Sex | *t*(90) = -2.496 | 0.602 | 0.014 |
| Group x Sex | *t*(90) = 0.442 | - | 0.660 |
